# Effects of *Eucommia ulmoides* Leaf Extract on Growth Performance, Serum Biochemistry, Rumen Microbiota, and Metabolic Profiles in Yaks

**DOI:** 10.3390/ani16182965

**Published:** 2026-09-20

**Authors:** Wenli Liu, Yan Yang, Yongfu La, Xiaoming Ma, Xiaoyun Wu, Min Chu, Xian Guo, Ping Yan, Chunnian Liang

**Affiliations:** 1 Lanzhou Institute of Husbandry and Pharmaceutical Sciences of Chinese Academy of Agricultural Sciences, Key Laboratory of Yak Breeding Engineering of Gansu Province, Lanzhou 730000, China; liuwenli0416@163.com (W.L.); 15103904873@163.com (Y.Y.); layongfu@caas.cn (Y.L.); maxiaoming@caas.cn (X.M.); wuxiaoyun@caas.cn (X.W.); chumin@caas.cn (M.C.); guoxian@caas.cn (X.G.); yanping@caas.cn (P.Y.); 2 Key Laboratory of Animal Genetics and Breeding on Tibetan Plateau, Ministry of Agriculture and Rural Affairs, Lanzhou 730000, China; 3 Institute of Animal Husbandry and Veterinary Medicine, Tibet Academy of Agriculture and Animal Husbandry Sciences, Lhasa 850004, China

**Keywords:** antioxidant function, *Eucommia* leaf extract, VFA, yaks

## Abstract

This study aimed to investigate the effects of adding *Eucommia ulmoides* leaf extract (ELE) to the diet on the growth performance, serum biochemical parameters, rumen fermentation parameters, rumen microorganisms and metabolites of yaks. Ten male yaks (two years old) were selected and randomly divided into two groups (*n* = 5). The control group (C, 153.48 ± 7.48 kg) was fed the basal diet, while the experimental group (H, 146.72 ± 1.87 kg) was fed the basal diet supplemented with 1.0 g/kg feed dry matter (DM) of ELE for 75 days. The results showed that the average daily gain, the serum total antioxidant capacity, catalase, and superoxide dismutase were significantly higher in the H group than in the C group (*p* < 0.01), while the serum total cholesterol, creatinine, alkaline phosphatase, blood urea nitrogen, and malondialdehyde were significantly lower than those of the C group (*p* < 0.05); in contrast, low-density lipoprotein cholesterol was significantly elevated in the H group (*p* < 0.01). Furthermore, the concentrations of acetate, butyrate, and hexanoic acid increased significantly compared with the C group (*p* < 0.05); 2-Hydroxypropanoic acid, butanedioic acid, and pentanedioic acid decreased significantly in the H group compared with the C group (*p* < 0.05). The metagenomic analysis revealed that ELE not only promoted the growth of cellulose-degrading bacteria but also significantly reduced the abundance of the virulence factors and carbohydrate-active enzymes (*p* < 0.05). In the experimental and control groups, the dominant microorganisms were *Bacillota* and *Prevotellaceae*, respectively. Metabolomics analysis further revealed that the differential metabolites were significantly enriched in pathways including purine metabolism, β-alanine metabolism, glutathione metabolism, and cGMP-PKG. The Pearson correlation analysis revealed that there were interactions among various microorganisms and metabolites, which can promote the growth and health of yaks. In conclusion, ELE can promote the growth and health of yaks by regulating the serum biochemical parameters, rumen fermentation, rumen microbial flora, and metabolism.

## Simple Summary

Yak farming is a economic mainstay for pastoral communities on the plateau, but the harsh climate often limits the animals’ growth and well-being. We tested whether adding a natural extract from *Eucommia ulmoides* leaves to their diet could help improve this situation. Our results showed that the extract boosted the yaks’ growth rate, reduced oxidative stress, and optimized the gut microbiota. Most importantly, it also lowered the levels of harmful toxins in the gut. This suggests that Eucommia leaf extract helps yaks grow healthier and faster, offering a practical way to increase farmers’ incomes.

## 1. Introduction

As the predominant livestock species endemic to the Qinghai–Tibet Plateau and adjacent high-altitude regions, the yak (*Bos grunniens*) serves as a cornerstone of both regional ecological stability and Tibetan cultural heritage. It provides indispensable livestock products, including meat, milk, fiber, and draught power—that sustain local pastoralist communities [1]. Notably, over 90% of the global yak population resides in China, where production systems are intrinsically linked to fragile alpine meadow and steppe ecosystems [1]. Yak husbandry is still characterized by traditional seasonal grazing, a practice that inherently limits productivity due to harsh environmental constraints and extensive management regimes [2]. Addressing the dual challenge of meeting growing market demands and enhancing the profitability of pastoralists requires a fundamental restructuring of the yak industry—moving away from mere numerical expansion toward a model emphasizing quality and efficiency. A pivotal component of this transformation involves the adoption of semi-grazing regimes coupled with sophisticated nutritional regulation. Through optimized ration formulation and strategic interventions, such as cold-season supplementation, it is possible to buffer environmental stressors and exploit untapped production potential. This approach represents a cornerstone for the long-term viability of the industry [3]. This has spurred interest in developing eco-friendly feed additives to boost yak health, feed efficiency, and product safety. Plant-based additives are particularly promising substitutes for antibiotic growth promoters, given their natural composition and broad-spectrum bioactivity. The leaves of *Eucommia ulmoides* Oliv., a tree indigenous to China, are abundant in functional components such as chlorogenic acid, flavonoids, and polysaccharides [4,5,6]. Notably, *Eucommia ulmoides* leaf extract (ELE) demonstrates comprehensive bioactivities, encompassing antimicrobial, anti-inflammatory, antioxidant, and immunomodulatory effects [5].

Accumulating evidence substantiates the beneficial effects of ELE in ruminant nutrition [4]. For instance, dietary inclusion of fermented ELE in beef feeding regimens has been shown to enhance growth performance and antioxidant capacity, while simultaneously improving the proximate composition and meat quality [7]. Furthermore, ELE supplementation appears to be enterically benign, as it does not adversely alter fecal microbial diversity but rather modulates the gut microbial architecture [8]. Consistent with this, studies in ovine models indicate that ELE-derived polyphenols optimize rumen fermentation dynamics by reducing ammonia nitrogen concentrations and shifting the profile of volatile fatty acids toward a lower acetic acid-to-propionic acid ratio (Ac:Pr) [9,10]. Based on the effective dose ranges reported in these studies [7,9,10] and the manufacturer’s recommendation, a supplementation level of 1.0 g/kg feed dry matter (DM) was selected for the present study. Collectively, these findings suggest a central mechanistic paradigm: ELE-mediated improvements in production performance and physiological health are intrinsically linked to the restructuring of the ruminal and intestinal microbiota, thereby orchestrating systemic metabolic homeostasis [4].

Yet, yaks represent a unique evolutionary trajectory; shaped by millennia of natural and artificial selection, they possess physiological idiosyncrasies tailored to high-altitude environments [11] and a specialized rumen ecosystem divergent from other domestic ruminants [12]. Crucially, the impact of ELE on the bidirectional signaling between the rumen microbiome and host metabolism in this sentinel species remains unexplored. Therefore, this study sought to decipher how dietary ELE supplementation influences growth performance, systemic health biomarkers, and the rumen environment. We posit that ELE acts as a modulator of the rumen ecosystem, restructuring microbial consortia and their metabolic outputs to facilitate efficient nutrient utilization and energy homeostasis. Ultimately, this research provides a mechanistic framework for leveraging ELE to bolster yak productivity and elucidates broader principles governing plant extract-mediated modulation of ruminant gastrointestinal ecology.

## 2. Materials and Methods

### 2.1. Experimental Animals and Management

All animal experimental procedures were approved by the Institutional Animal Care and Use Committee (IACUC) of the Lanzhou Institute of Animal Husbandry and Pharmaceutical Sciences, Chinese Academy of Agricultural Sciences (CAAS) (approval number: 1610322020018) and strictly adhered to the Guidelines for the Ethical Handling of Farm Animals in China. The experiment was conducted at the Liqiyalu Ranch in Gannan County, Gansu Province, China. Situated in a typical high-altitude yak farming region, this site was characterized by a cold climate and hypoxic conditions. This environment provides a representative setting for investigating the adaptive physiology and metabolic responses of yaks to nutritional interventions. Ten male yaks, approximately two years of age with similar body weight (BW ± SEM) and in good health, were selected and randomly divided into two groups (*n* = 5, one pen per group) based on similar BW using R 4.1.2 software. The basal diet was formulated as a concentrate supplement based on the Feeding Standard of Beef Cattle (NY/T 815-2004) [13] to meet the basal physiological nutrient requirements of two-year-old yaks. The chemical composition of each ingredient was referenced from the Chinese Feed Database (2023) [14] and Feedipedia (2024) [15]. The dietary ingredient composition and nutrient levels are presented in Table 1. The control group (C, initial BW: 153.48 ± 7.48 kg) was fed a basal diet (Table 1), while the ELE group (H, initial BW: 146.72 ± 1.87 kg) was fed the same basal diet supplemented with 1.0 g/kg feed DM of ELE on top of the basal ration (dry matter basis). The experiment lasted 75 days, including a 15 d adaptation period (11–25 August 2026) and a 60 d experimental period. The initial body weight (IBW) of all yaks was measured before the formal period. All yaks were weighed at three time points (initial, day 30, and final) before the morning feeding (08:00 h) following an overnight fast. All yaks were group-housed and group-fed under a uniform feeding regimen, with concentrating supplementary diets provided twice a day at 8:00 h and 16:00 h, allowing ad libitum intake of concentrate supplementary diets, oat straw and water. The testing equipment was cleaned and disinfected regularly, and all other management and disease prevention measures also followed the regular procedures of the farm.

### 2.2. Source and Composition of Eucommia Leaf Extract

The ELE powder was purchased from Zhangjiajie Hengxing Biotechnology Co., Ltd. (Zhangjiajie, China). According to the manufacturer, the product contains chlorogenic acid (5%), flavonoids (8%), and polysaccharides (20%), which is consistent with the reported phytochemical profile of *Eucommia ulmoides* leaves [4].

### 2.3. Blood Sample Collection and Analyses

At the end of the experiment, blood samples were aseptically collected from the jugular vein of all yaks into vacuum tubes containing a clotting activator. The samples were maintained at 4 °C for 4 h to facilitate coagulation and then centrifuged at 1500× *g* for 15 min at 4 °C. The resulting serum supernatants were carefully aspirated and stored at −20 °C pending analysis of biochemical parameters. Serum concentrations of total cholesterol (TC), high-density lipoprotein cholesterol (HDL-C), low-density lipoprotein cholesterol (LDL-C), creatinine (Cr), blood urea nitrogen (BUN), alkaline phosphatase (AKP), aspartate aminotransferase (AST), total antioxidant capacity (T-AOC), malondialdehyde (MDA), glutathione peroxidase (GSH-Px), superoxide dismutase (SOD), and catalase (CAT) were quantified using standardized spectrophotometric and ELISA methods. Biochemical assays were conducted using kits from Nanjing Jiancheng Bioengineering Institute (Nanjing, China), and ELISA kits were sourced from ELK Biotechnology (Wuhan, China), strictly adhering to the provided instruction manuals. All measurements were performed in duplicate to confirm accuracy.

### 2.4. Rumen Fluid Sampling and Analyses

For sampling, experimental yaks were gently guided into a designated handling chute and physically restrained using a squeeze chute. Local anesthesia was achieved by subcutaneous infiltration of 2% lidocaine hydrochloride at the left paralumbar fossa. Following disinfection with 75% ethanol, rumen fluid was collected via percutaneous trocarisation using a sterile cannula (length: 180–200 cm; outer diameter: 16–18 mm). The cannula was inserted vertically into the ventral sac of the rumen and connected to a vacuum pump (Profs Products, Visbek, Germany). Upon opening the valve, fluid flowed spontaneously; the initial 50 mL was discarded to flush the cannula and minimize contamination from rumen contents and particulate matter introduced during trocar placement. The collected fluid was subsequently filtered through four layers of sterile cheesecloth and centrifuged at 2000× *g* for 10 min at 4 °C. The resulting supernatant and pellet were immediately divided into aliquots and stored at −80 °C until analysis by UPLC-ESI-MS/MS. The supernatant was used for metabolomics and volatile fatty acid (VFA) determination, while the pellet was used for microbial DNA extraction. For metabolomic preparation, 80 µL aliquots of the supernatant were subjected to derivatization with a cocktail comprising 40 µL of 200 mM 3-nitrophenylhydrazine (3-NPH) and 40 µL of 120 mM 1-ethyl-3-(3-dimethylaminopropyl) carbodiimide (EDC) in 6% pyridine at 40 °C for 30 min, followed by immediate cooling on ice. All samples were ultimately preserved at −80 °C until analysis by UPLC-ESI-MS/MS. Ruminal VFA concentrations were determined based on the untargeted metabolomics data; individual volatile fatty acids (VFAs) (acetic, propionic, butyric, valeric, and hexanoic acids) were identified by matching retention times and MS/MS fragmentation spectra against the database, and quantified from chromatographic peak areas.

### 2.5. Metagenomic Sequencing and Bioinformatics Analyses

Total genomic DNA was extracted from rumen fluid microbial samples using the QIAamp Fast DNA Mini Kit (Qiagen, Hilden, Germany). The concentration and purity of the extracted DNA were quantified using a NanoDrop 2000 spectrophotometer (Thermo Fisher Scientific, Waltham, MA, USA), while DNA integrity was assessed via 1% agarose gel electrophoresis. Subsequent purification steps were performed to remove residual impurities and small-sized fragments. Finally, a sequencing library was constructed using the TruSeq Nano DNA LT Library Prep Kit (Illumina, San Diego, CA, USA) following the manufacturer’s protocol. The qualified library was sequenced on the Illumina NovaSeq 6000 platform, generating 150 bp paired-end reads. Raw sequence data were quality-filtered using fastp (v0.23.4; Shifu Chen, HaploX Biotechnology/OpenGene, Shenzhen, China) [16]. Adapter sequences and low-quality reads were trimmed, and the resulting high-quality clean reads were statistically summarized. Quality control metrics, including raw and effective read counts, Q30 scores, and GC content, were comprehensively evaluated to ensure dataset integrity prior to downstream analysis. To mitigate host DNA contamination, quality-filtered reads were aligned to the yak (*Bos grunniens*) reference genome using BBMap (v39.01; Brian Bushnell, Oregon State University, Corvallis, OR, USA) [17], and non-host sequences were extracted for downstream analysis. Subsequently, metagenomic assembly was performed on the filtered reads to reconstruct longer contigs. This step was critical to maximize the recovery of genomic information and to ensure the accuracy of subsequent functional annotation. Metagenomic assembly of the host-filtered clean reads was conducted using MEGAHIT (v1.2.9; Center for Bioinformatics, Peking University, Beijing, China) [18], a de Bruijn graph-based assembler that constructs graph structures by leveraging overlaps between k-mers to generate contiguous sequences (contigs). Contigs ≥ 500 bp in length were retained, enumerated, and utilized for all subsequent bioinformatic analyses. Following assembly, open reading frames (ORFs) were predicted from the resulting contigs using Prodigal (v2.6.3; Oregon State University, Corvallis, OR, USA) [19], and the identified sequences were subsequently translated into amino acid sequences. For ORF predictions derived from both individual samples and the co-assembly, MMseqs2 (v14-7e284; MPI for Developmental Biology, Tübingen, Germany) [20] was used to dereplicate gene catalogs and generate a non-redundant unigene set. Clustering was performed using CD-HIT with default parameters (95% identity, 90% coverage), selecting the longest sequence in each cluster as the representative. A Salmon index was subsequently built based on this non-redundant gene set. Using Salmon (v1.10.0; COMBINE Lab, University of California, Riverside, CA, USA) [21], clean reads from each sample were quasi-mapped to the index to quantify gene abundances. Low-abundance genes failing to meet a minimum read count threshold of 2 across all samples were filtered out to yield the final unigene set. Gene abundance tables were normalized based on read counts and gene lengths. Furthermore, taxonomic assignment was performed by aligning the unigenes against the NCBI NR database using BLASTP (v2.14.1; National Center for Biotechnology Information, Bethesda, MD, USA). The relative abundance of taxa was subsequently profiled at various taxonomic ranks, including phylum, class, order, family, genus, and species, to construct comprehensive community profiles.

### 2.6. Metabolomics Measurement and Bioinformatics Analyses

Metabolite profiling was performed using a UHPLC-Q Exactive HF-X mass spectrometer (Thermo Fisher Scientific, Waltham, MA, USA). Briefly, samples stored at −80 °C were thawed on ice. A 100 µL aliquot was mixed with 400 µL of pre-chilled protein precipitation solvent (methanol: acetonitrile = 2:1, *v*/*v*) containing a 4 µg/mL internal standard mixture. After brief vortexing, samples were subjected to ice-bath sonication for 10 min, followed by centrifugation at 12,000× *g* for 20 min at 4 °C. The resulting supernatants (150 µL) were carefully transferred into glass autosampler vials for LC-MS analysis. Quality control (QC) samples, prepared by pooling equal volumes of all extracts, were interspersed throughout the run to monitor system stability and data reproducibility. Chromatographic separation was performed on a Waters ACQUITY UPLC I-Class PLUS system (Waters Corporation, Milford, MA, USA) coupled to a Thermo Q Exactive™ HF-X hybrid quadrupole-Orbitrap mass spectrometer (Thermo Fisher Scientific, Waltham, MA, USA). Separation was achieved using an ACQUITY UPLC HSS T3 column (100 mm × 2.1 mm i.d., 1.8 µm) maintained at 45 °C. The mobile phase consisted of 0.1% formic acid in water (A) and acetonitrile (B), with a flow rate of 0.35 mL/min. The injection volume was set to 3 µL. Mass spectrometry data were acquired in both positive and negative electrospray ionization (ESI) modes. The spray voltage was set at ±3.5 kV. Full MS scans were collected at a resolution of 70,000 (FWHM at *m*/*z* 200) over a mass range of *m*/*z* 70–1050. To monitor system stability, a pooled QC sample was injected after every 10 analytical runs. Raw data were processed using Progenesis QI software (version 2.3; Waters Corporation, Milford, MA, USA). A three-dimensional data matrix comprising retention time, *m*/*z*, and ion intensity was generated. Features with >20% missing value across all samples were removed. The remaining missing values were imputed with the minimum observed value. Finally, data normalization was performed using the sum of intensities to correct for potential dilution effects.

### 2.7. Calculations, Data Processing and Analyses

The average daily gain (ADG = (Final average weight − Initial average weight)/Number of days in the experiment) was calculated. Statistical analyses were performed using IBM SPSS Statistics for Windows (Version 26.0; IBM Corp., Armonk, NY, USA). All variables, including growth performance (Table 2), serum biochemical parameters (Table 3), rumen volatile fatty acids (Table 4), rumen microbiota, and metabolomes, were analyzed based on the randomly selected sub-sample described in Section 2.3 (*n* = 5 per group). The individual yak was considered the experimental unit for all statistical analyses. Data are presented as mean ± standard error of the mean (SEM). Normality was assessed visually using histograms and Q-Q plots. Homogeneity of variances was formally tested using Levene’s test. Given the limited sample size (*n* = 5 per group), normality was assessed primarily through graphical methods (histograms and Q-Q plots), as formal normality tests have low statistical power with small samples. Independent samples *t*-tests were used for growth performance, serum biochemistry, and VFA data, as *t*-tests are relatively robust to mild departures from normality with equal sample sizes. For microbiome compositional data, the non-parametric Wilcoxon rank-sum test was used as described in the respective sections. Group comparisons were conducted using independent samples *t*-tests. For parameters with homogeneous variances (including ADG; Levene’s *p* > 0.05), per-group SEM was reported for each group. Where Levene’s test indicated heterogeneous variances (if any), Welch’s correction was applied. Effect sizes were calculated as Cohen’s d for all pairwise comparisons; values of |d| ≥ 0.8 were interpreted as large effects. A two-tailed *p* < 0.05 was considered statistically significant. For microbiome analysis, the relative abundance of genes and taxa at various phylogenetic levels was calculated based on read counts and sequence lengths. Multivariate statistical analyses, including Principal Component Analysis (PCA) and Partial Least Squares Discriminant Analysis (PLS-DA), were performed using SIMCA-P software (v13.0; Sartorius Stedim Data Analytics AB, Umeå, Sweden) [22] to visualize sample clustering and discriminate between experimental groups. Model robustness was evaluated via seven-fold cross-validation and 200 response permutation tests (RPT) to guard against overfitting. Differential abundance of microbial features—including Kyoto Encyclopedia of Genes and Genomes (KEGG) pathways, Carbohydrate-Active enZYmes (CAZy) families, virulence factors (VFDB), and antibiotic resistance genes (CARD)—was identified using Linear Discriminant Analysis Effect Size (LEfSe), with significance defined as a linear discriminant analysis (LDA) score > 2 and a false discovery rate (FDR) adjusted *p* < 0.05.

For metabolomic data, QC was implemented by retaining metabolite features with a relative standard deviation (RSD) ≤ 30% in pooled QC samples. Differential metabolites were screened using the Wilcoxon rank-sum test, with multiple testing corrections applied via the Benjamini–Hochberg (BH) procedure (*p* < 0.05). Volcano plots were generated using R software (v4.1.2; R Foundation for Statistical Computing, Vienna, Austria) [23] and the ggplot2 package (v3.3.5; Hadley Wickham, RStudio, Boston, MA, USA) [24]. To elucidate inter-kingdom associations, Pearson correlation analysis was conducted between phenotypic parameters, differential metabolites, and microbial features (Variable Importance in Projection, VIP > 2). Pearson correlation coefficients (r) and the corresponding two-tailed *p*-values were computed for each pair of variables. To visualize the correlation structure, cluster heatmaps were generated using Origin Ro 2025 (v9.9.0; Origin Lab Corp, Northampton, MA, USA) [25], with hierarchical clustering applied independently to both rows and columns based on Euclidean distance and average linkage (UPGMA). Given the total sample size of *n* = 10, correlations were considered statistically significant at *p* ≤ 0.05 (corresponding to |r| ≥ 0.63), and only significant correlations were retained in the heatmaps. Correlation strength was interpreted as very strong (|r| ≥ 0.8), strong (0.6 ≤ |r| < 0.8), moderate (0.4 ≤ |r| < 0.6), or weak (0.2 ≤ |r| < 0.4).

## 3. Results

### 3.1. Effect of ELE on the Growth Performance of Yaks

As shown in Table 2, there were no significant differences in IBW, 30-day BW, or final body weight (FBW) between the two groups (*p* > 0.05). Conversely, the ADG of the H group was significantly higher than that of the C group (*p* < 0.01). Levene’s test confirmed homogeneous variances for all growth parameters (*p* > 0.05); thus, independent samples *t*-tests with per-group SEM were applied.

### 3.2. Effect of ELE on the Blood Parameters of Yaks

As shown in Table 3, dietary supplementation with ELE significantly enhanced serum T-AOC, CAT, and SOD levels in yaks (*p* < 0.05), with T-AOC and CAT exhibiting marked elevation (*p* < 0.01). Conversely, ELE supplementation significantly reduced the concentrations of LDL-C, MDA, Cr, AKP, BUN, and TC (*p* < 0.05). Notably, LDL-C and BUN showed the most pronounced changes (*p* < 0.01). No significant alterations were observed in HDL-C, AST, or GSH-Px levels (*p* > 0.05).

**Table 3:** Effect of ELE on the blood parameters of yaks (*n* = 5).

Items	Reference Values	Groups		*p*-Value
		C Group	H Group	
High-density lipoprotein cholesterol (HDL-C), mmol/L	1.40–3.10	2.16 ± 0.23	2.22 ± 0.14	0.824
Low-density lipoprotein cholesterol (LDL-C), mmol/L	0.02–0.80	0.30 ± 0.02	0.50 ± 0.04	0.004
Total antioxidant capacity (T-AOC), U/mL	5.00–10.00	7.88 ± 0.45	10.42 ± 0.14	<0.010
Catalase (CAT), U/mL	8.00–15.00	11.54 ± 0.26	16.07 ± 0.54	<0.010
Malondialdehyde (MDA), nmol/mL	2.00–5.00	3.00 ± 0.22	1.75 ± 0.24	0.005
Creatinine (Cr), μmol/L	40.00–125.00	90.40 ± 1.25	70.76 ± 3.55	<0.010
Alkaline phosphatase (AKP), King-Armstrong unit/100 mL	2.00–4.00	3.04 ± 0.21	2.20 ± 0.26	0.037
Blood urea nitrogen (BUN), mmol/L	1.30–9.70	6.22 ± 0.33	4.43 ± 0.23	0.002
Total cholesterol (TC), mmol/L	1.10–3.50	3.03 ± 0.24	2.10 ± 0.28	0.035
Aspartate aminotransferase (AST), U/L	40.00–130.00	73.77 ± 7.00	70.25 ± 6.13	0.715
Glutathione peroxidase (GSH-Px), U/mL	90.00–420.00	402.78 ± 24.08	459.83 ± 22.15	0.119
Superoxide dismutase (SOD), U/mL	55.00–100.00	53.17 ± 2.94	68.02 ± 3.45	0.011

Notes: Values are analyzed by independent samples *t*-tests and presented as mean ± SEM. Statistical significance was set as *p* < 0.05. Reference values are sourced from published literature and assay kit manuals: TC, BUN, Cr, and AST from Siachos et al. [26]; HDL-C and LDL-C from Yang et al. [27]; SOD and GSH-Px from Wu et al. [28]; T-AOC and MDA from Zhu et al. [29]; AKP and CAT from the assay kit instruction manuals (Nanjing Jiancheng Bioengineering Institute, Nanjing, China).

### 3.3. Effect of ELE on the VFA of Yaks

As shown in Table 4, ELE supplementation significantly altered several volatile fatty acid profiles in yaks. Compared with the C group, the H group exhibited elevated concentrations of acetate (*p* < 0.01), butyrate (*p* < 0.01), and hexanoic acid (*p* < 0.01). Conversely, butanedioic acid (*p* < 0.01) and pentanedioic acid *(p* < 0.05) were significantly reduced in the H group. 2-Hydroxypropanoic acid was also significantly lower in the H group (*p* < 0.05). Propionate showed a numerical decrease in the H group (*p* > 0.05) with a very large effect size, suggesting that the failure to reach statistical significance likely reflects the limited sample size rather than the absence of a biologically meaningful effect. Additionally, the H group exhibited a significantly higher total VFA concentration (*p* < 0.05) and a higher acetate to propionate ratio (Ac:Pr) (*p* < 0.001) compared with the C group.

**Table 4:** Effect of ELE on the VFA of yaks (*n* = 5).

Items (mmol/L)	Groups		Cohen’s D	*p*-Value
	C Group	H Group		
Acetate	12.21 ± 0.39	14.61 ± 0.32	−3.00	0.001
Propionate	3.90 ± 0.17	3.49 ± 0.05	+1.45	0.051
Butyrate	2.12 ± 0.09	2.55 ± 0.08	−2.28	0.007
Valerate	0.45 ± 0.02	0.40 ± 0.01	+1.19	0.097
Hexanoic acid	0.14 ± 0.03	0.38 ± 0.04	−3.40	<0.010
Isobutyrate	0.46 ± 0.03	0.48 ± 0.03	−0.30	0.691
Isovalerate	0.36 ± 0.02	0.34 ± 0.01	+0.67	0.349
2-Hydroxypropanoic acid	0.32 ± 0.10	0.02 ± 0.01	+1.88	0.018
Butanedioic acid	0.10 ± 0.02	0.01 ± 0.00	+2.67	<0.010
Pentanedioic acid	0.08 ± 0.02	0.01 ± 0.00	+1.85	0.019
Total VFA	19.47 ± 0.51	22.16 ± 0.38	−2.66	0.003
Ac:Pr	3.15 ± 0.08	4.20 ± 0.15	−3.93	<0.001

Notes: All values were analyzed by independent-sample *t*-tests and presented as mean ± SEM. Statistical significance was set as *p* < 0.05. Variables (pooled-variance, df = 8), except isovalerate (Welch’s *t*-test, C *n* = 4 vs. H *n* = 5) and isobutyrate (df = 6, *n* = 4 per group) due to samples that failed QC. Cohen’s d = (M_1_ − M_2_)/SD_pooled; positive values indicate higher concentrations in the C group.

### 3.4. Effect of ELE on the Rumen Microorganisms of Yaks

To characterize the effects of ELE on the yak rumen microbiota, we generated metagenomic sequencing data from 10 rumen fluid samples (5 per group). A total of 79,765,743 and 79,735,634 raw reads were recovered from the control and supplemented groups, respectively. Following quality filtering and depletion of host-genome sequences, 78,395,036 and 78,351,817 clean reads were retained for downstream analysis, with Q30 bases accounting for >95.09% of total reads in both groups. Retained clean reads were assembled de novo, yielding average counts of 317,226.6 and 441,568.8 per sample in the C and H groups, respectively, with mean GC contents of 45.36% and 44.38%. The average N50 and N90 values across all samples were 1654.3 bp and 593.8 bp, respectively. Subsequent gene prediction identified 2,646,552 and 3,592,658 ORFs in the C and H groups, respectively (Tables S1–S3).

**Figure 1:**
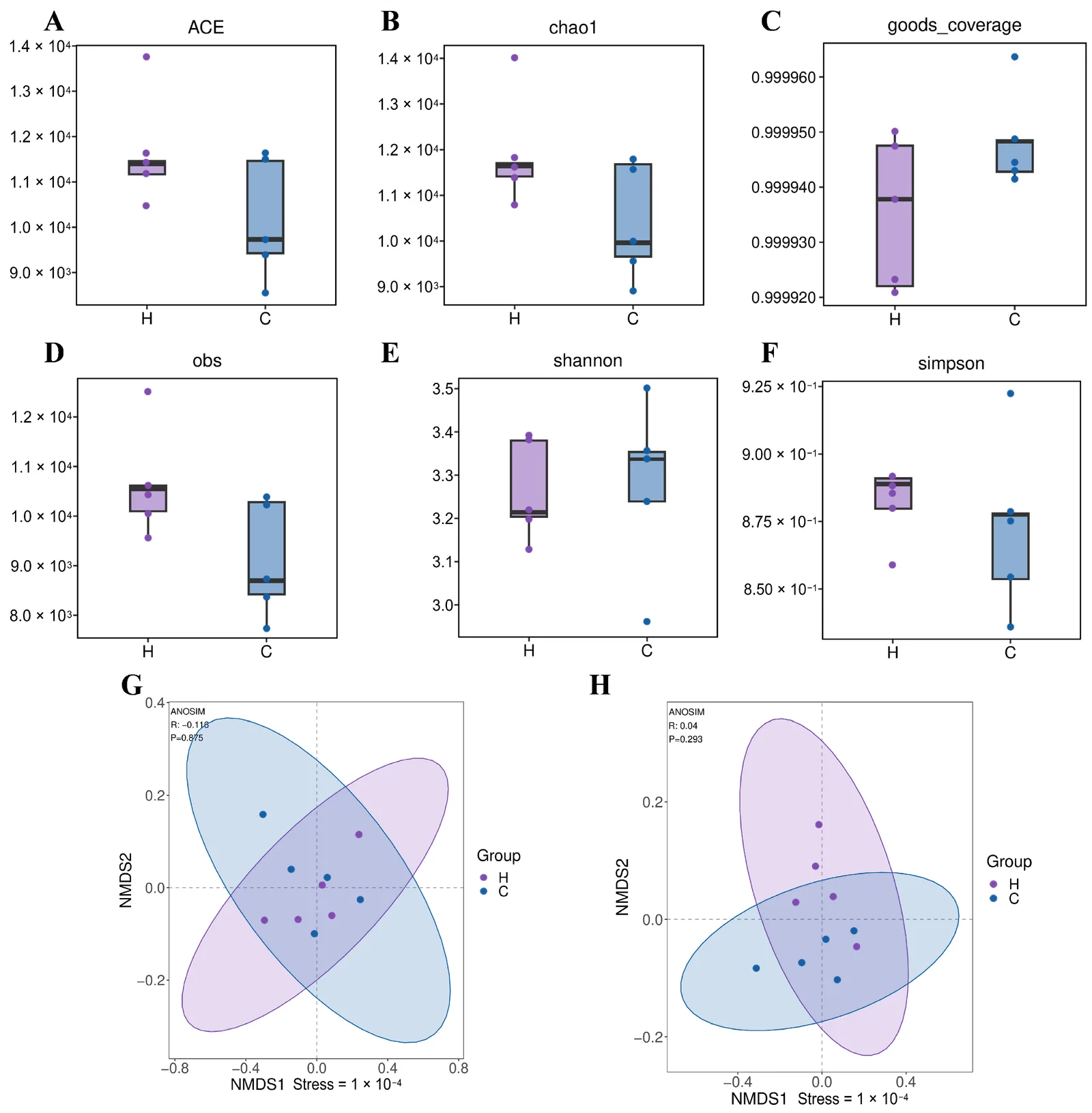
Diversity indices of the rumen microbial community in yaks. (**A**) ACE, (**B**) Chao1, (**C**) Good’s coverage, (**D**) Observed species, (**E**) Shannon, (**F**) Simpson. NMDS analyses at the phylum and genus levels are shown in panels (**G**,**H**). In panels (**A**–**F**), each point represents an individual yak (*n* = 5 per group) and the shaded areas indicate the data distribution within each group; blue denotes the C group and purple denotes the H group. In panels (**G**,**H**), each point represents one sample.

To characterize the effect of ELE on the rumen microbiome, we evaluated both α- and β-diversity metrics. For α-diversity, boxplots (Figure 1A–F) revealed no statistically significant differences between the control (C) and supplemented (H) groups across the ACE, Chao1, Observed species (Obs), Shannon, Simpson, and Good’s coverage indices. Notably, however, visual inspection revealed a non-significant trend toward higher richness in the H group, as evidenced by elevated median values for Obs (Figure 1D) and Simpson (Figure 1F) indices relative to the C group. β-diversity analysis via non-metric multidimensional scaling (NMDS) ordination (Figure 1G,H) demonstrated clear segregation of samples by treatment group along the first two axes. At both the phylum and genus taxonomic levels, samples exhibited tight intra-group cohesion and distinct inter-group separation, with replicate samples clustering closely together. The C group formed a compact, homogeneous cluster, whereas H group samples were more diffusely distributed, suggesting that dietary ELE modulated rumen microbiome composition and increased inter-individual compositional variability within the treatment group.

**Figure 2:**
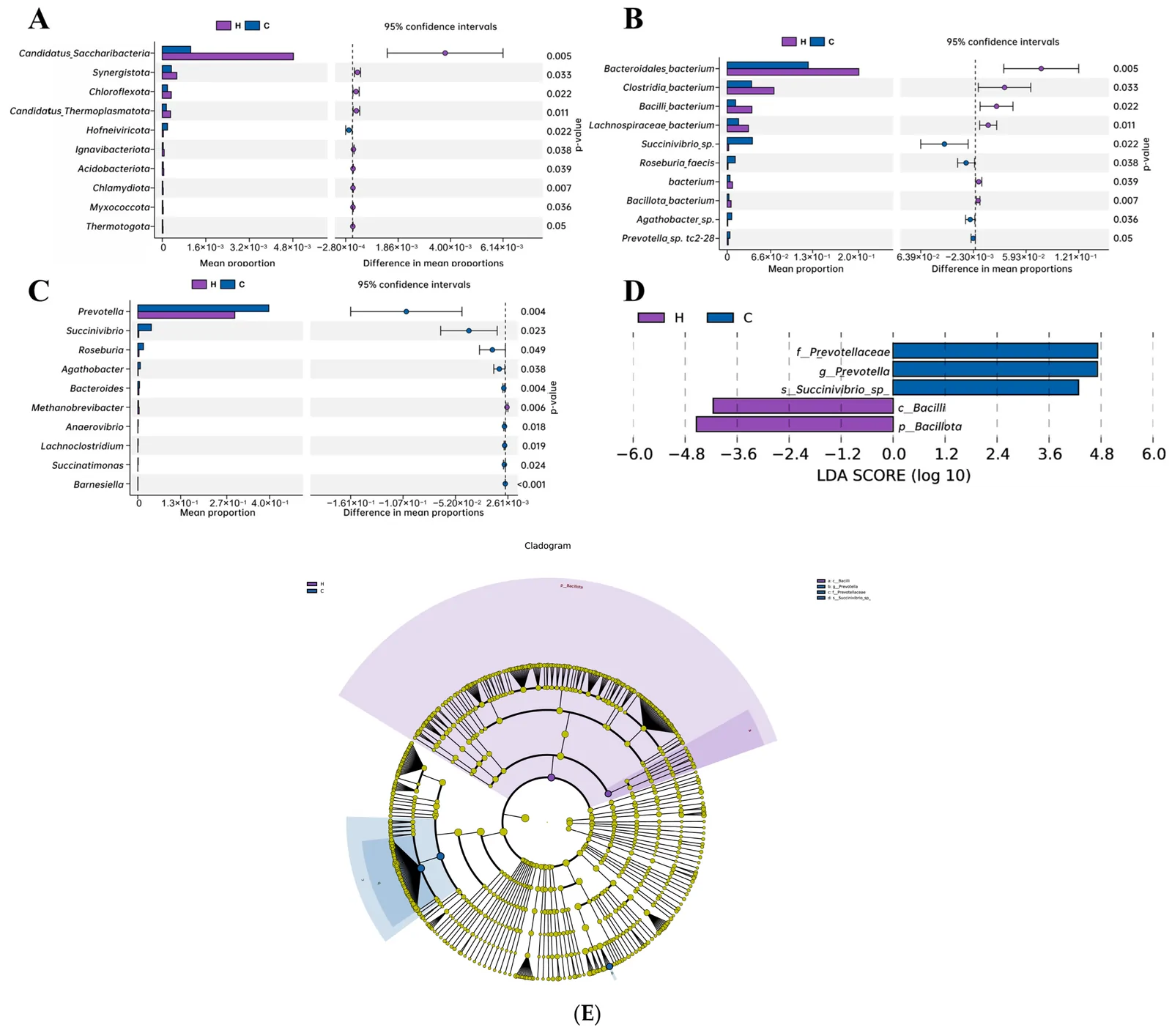
Rumen microbial community structure and taxonomic composition in yaks. The Wilcoxon rank-sum test was performed to compare species abundance between groups at the phylum (**A**), genus (**B**), and species (**C**) levels. The corresponding *p*-values are displayed. (**D**) Score plot of differentially abundant species. (**E**) Phylogenetic tree illustrating the annotation branches of differential species. In panels (**A**–**D**), horizontal bars represent the relative abundance of each taxon in the C (blue) and H (purple) groups. In panel (**D**), bars indicate the scores of differentially abundant species. In panel (**E**), branches and nodes colored blue or purple denote differentially abundant taxa enriched in the corresponding group.

At the phylum level (Figure 2A), differential abundance analysis using the Wilcoxon rank-sum test revealed distinct taxonomic shifts between the two groups. *Hofneiviricota* was significantly enriched in the control (C) group (*p* < 0.05). In contrast, *Candidatus saccharibacteria*, *Synergistota*, *Chloroflexota*, *Candidatus thermoplasmatota*, *Ignavibacteriota*, *Acidobacteriota*, *Chlamydiota*, *Myxococcota*, and *Thermotogota* exhibited significantly higher relative abundances in the supplemented (H) group (*p* < 0.05). Notably, *Acidobacteriota* demonstrated the most pronounced divergence, with an exceptionally high significance level (*p* < 0.001). At the genus level (Figure 2B), differential abundance analysis revealed distinct community shifts between the two treatment groups. *Methanobrevibacter* was significantly enriched in the supplemented (H) group (*p* < 0.05). Conversely, *Prevotella*, *Succinivibrio*, *Roseburia*, *Agathobacter*, *Bacteroides*, *Anaerovibrio*, *Lachnoclostridium*, *Succinatimonas*, and *Barnesiella* exhibited significantly higher relative abundances in the control (C) group (*p* < 0.05). Notably, *Barnesiella* demonstrated the most pronounced inter-group divergence, reaching exceptional statistical significance (*p* < 0.001). At the species level (Figure 2C), *Bacteroidales bacterium*, *Clostridia bacterium*, *Bacilli bacterium*, *Lachnospiraceae bacterium*, *Bacterium*, *Bacillota bacterium* were significantly enriched in the H group (*p* < 0.05), whereas *Succinivibrio* sp., *Roseburia faecis*, *Agathobacter* sp., *Prevotella* were significantly enriched in the C group (*p* ≤ 0.05). LEfSe analysis indicated significant differentiation in the composition of rumen microbial communities between the H group and the C group; the H group was significantly enriched in p_*Bacillota* and c_*Bacilli*, while the C group was significantly enriched in f_*Prevotellaceae*, g_*Prevotella*, and s_*Succinivibrio* sp. (Figure 2D,E).

**Figure 3:**
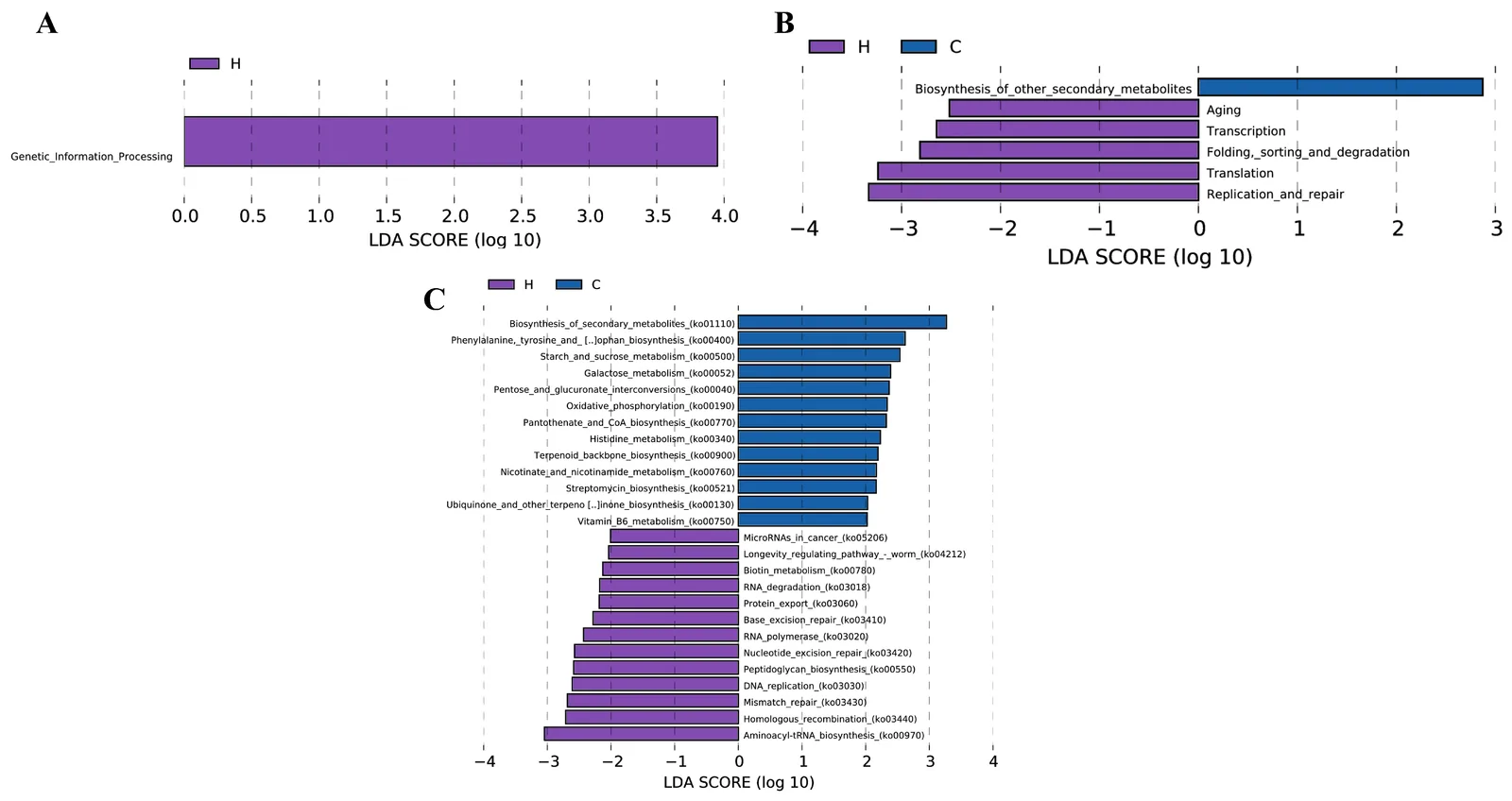
Functional differences in the rumen microbiota of yaks based on LEfSe analysis. LDA score distributions for features exceeding the significance threshold are presented at Level 1 (**A**), Level 2 (**B**), and Level 3 (**C**).

Functional pathway analysis revealed significant differences in the relative abundance of microbial metabolic pathways between the H and C groups. At KEGG Level 1 (Figure 3A), genetic information processing was enriched in the H group. At Level 2 (Figure 3B), aging, transcription, folding, sorting and degradation, translation, and replication and repair were significantly enriched in the H group (*p* < 0.05), whereas biosynthesis of other secondary metabolites was significantly enriched in the C group (*p* < 0.05). At KEGG Level 3 (Figure 3C), the C group was predominantly represented by diverse metabolic branches, particularly pathways related to carbohydrate and energy metabolism [Biosynthesis of secondary metabolites (ko01110), Phenylalanine, tyrosine and tryptophan biosynthesis (ko00400), Starch and sucrose metabolism (ko00500), Galactose metabolism (ko00052), Pentose and glucuronate interconversions (ko00040), Oxidative phosphorylation (ko00190), Pantothenate and CoA biosynthesis (ko00770), Histidine metabolism (ko00340), Terpenoid backbone biosynthesis (ko00900), Nicotinate and nicotinamide metabolism (ko00760), Streptomycin biosynthesis (ko00521), Ubiquinone and other terpenoid-quinone biosynthesis (ko00130), Vitamin B6 metabolism (ko00750)] (*p* < 0.05). The H group was mainly enriched in pathways related to DNA repair, transcription, and translation mechanisms [MicroRNAs in cancer (ko05206), Longevity regulating pathway-worm (ko04212), Biotin metabolism (ko00780), RNA degradation (ko03018), Protein export (ko03060), Base excision repair (ko03410), RNA polymerase (ko03020), Nucleotide excision repair (ko03420), Peptidoglycan biosynthesis (ko00550), DNA replication (ko03030), Mismatch repair (ko03430), Homologous recombination (ko03440), Aminoacyl-tRNA biosynthesis (ko00970)] (*p* < 0.05).

**Figure 4:**
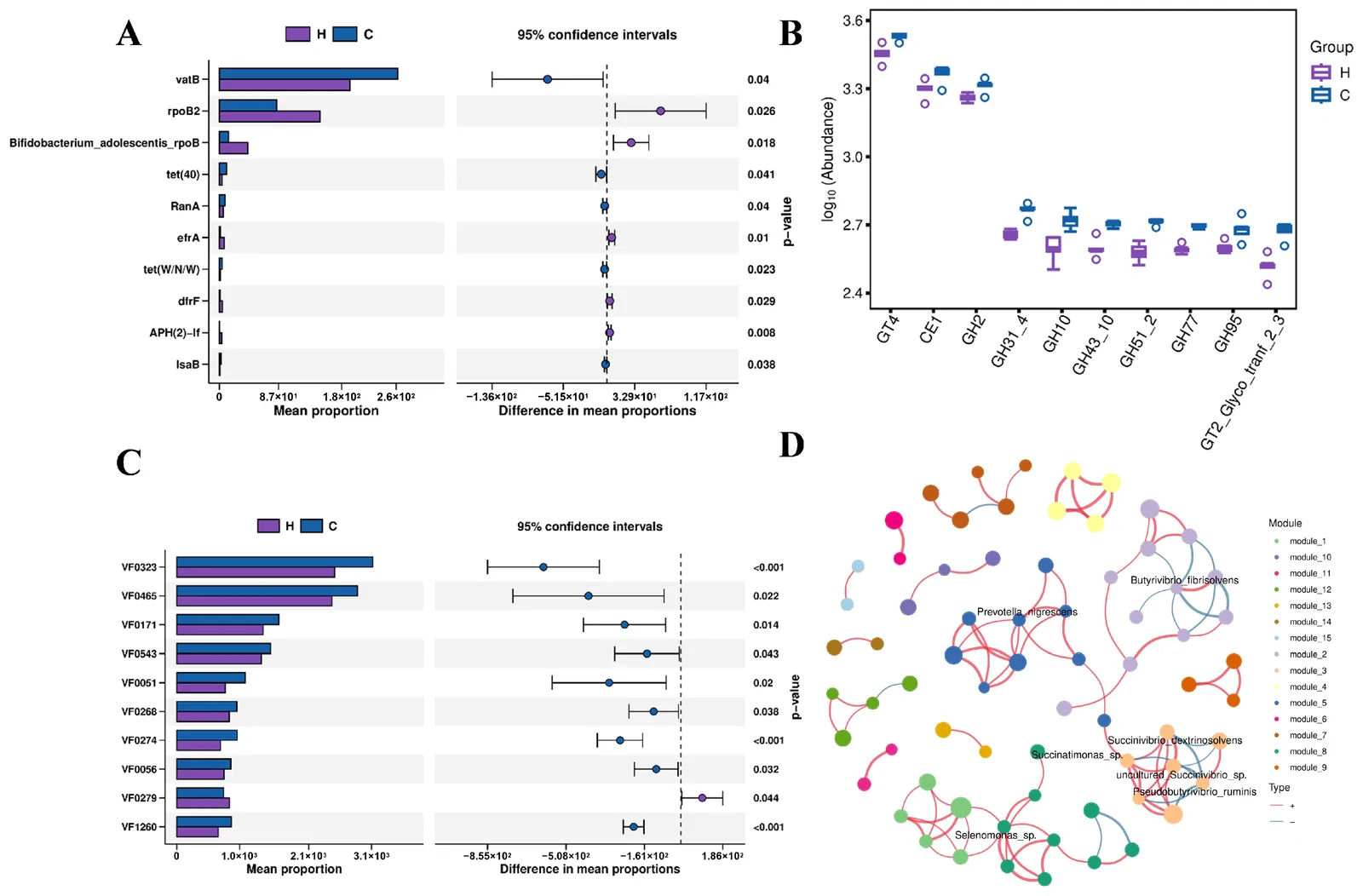
Functional annotation and abundance comparison of the rumen microbiota in yaks. (**A**) ARO-based antibiotic resistance gene abundance. (**B**) Significant CAZy family boxplots. (**C**) VFDB differential abundance bar charts. (**D**) Feature correlation network showing the interaction relationships of rumen microorganisms at the genus level. Blue and purple denote the C and H groups, respectively.

This study further analyzed the CAZy, VFDB, and CARD annotations at the family level. The CARD analysis (Figure 4A) indicated that in the C group, vat B, tet(40), RanA, tet(W/N/W), and isaB were significantly higher than in the H group (*p* < 0.05), while in the H group, rpoB2, rpoB, efrA, dfrF/A, and APH (2)-If were significantly higher than in the C group (*p* < 0.05). At the abundance level of the CAZy family (Figure 4B), the high-abundance families (GT4, CE1, GH2) had overall abundances in the H group similar to or slightly higher than those in the C group, with small differences in abundance between the groups; for the medium to low-abundance CAZy families (GH31_4, GH10, GH43_10, GH51_2, GH77, GH95, GT2_Glyco_tranf_2_3), the overall abundance in the H group was generally lower than that in the C group, indicating that the relative abundance of these carbohydrate-active enzyme families in the C group was higher. The VFDB analysis (Figure 4C) indicated that the abundance of all the displayed virulence factors was significantly higher in the C group than in the H group (*p* < 0.05); in the H group, there were no significantly enriched virulence factors. Finally, Figure 4D presents the interaction relationships of rumen microorganisms at the genus level. The analysis shows that multiple core microbial groups and their module affiliations interact with each other: *Prevotella nigrescens* has a strong correlation with module 5 and module 2, indicating that they play a crucial connecting role in the community structure. *Butyrivibrio fibrisolvens* have a strong association with module 2, *Succinivibrio dextrinosolvens*, uncultured *Succinivibrio* sp., *Pseudobutyrivibrio ruminis* have a strong association with module 13, *Succinatimonas* sp. *and Selenomonas* sp. have a strong association with modules 8 and 12. These organisms form relatively independent sub-networks, indicating their local dominance in specific metabolic functions.

### 3.5. Effect of ELE on the Rumen Metabolites of Yaks

**Figure 5:**
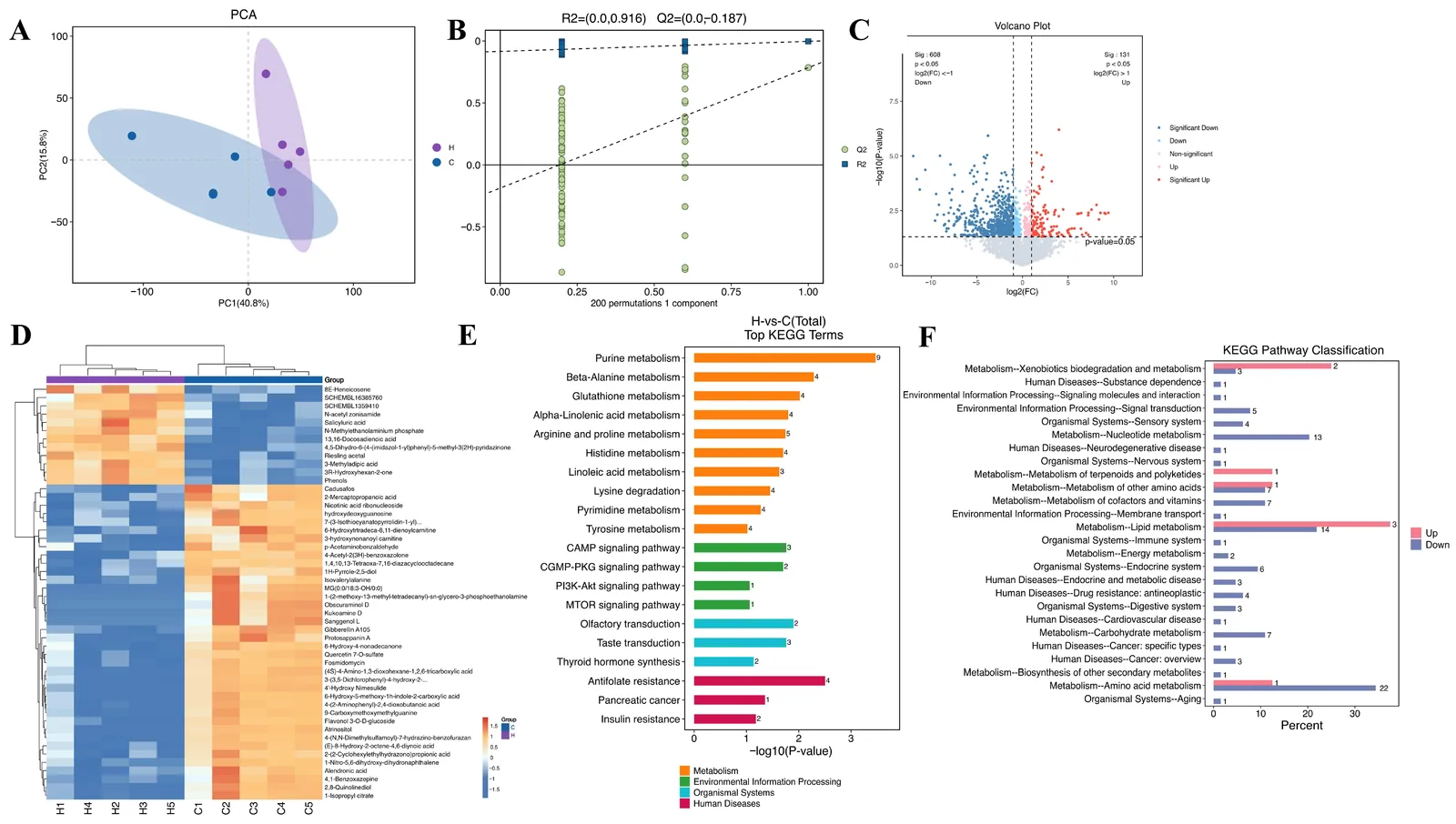
Impact of ELE on the rumen metabolome in yaks. (**A**) PCA score plot. (**B**) Model validation via permutation test. (**C**) Volcano plot of differential metabolites. (**D**) Heatmap of the top 50 differential metabolites. (**E**) Top 20 KEGG pathways. (**F**) Distribution of KEGG Level 2 pathways. In panels (**A**,**B**), blue denotes the C group and purple denotes the H group. In panel (**C**), red indicates upregulated metabolites and blue indicates downregulated metabolites.

To further analyze the differences in the microbial communities between the H group and the C group, this study analyzed them from the rumen metabolic perspective. In PCA (Figure 5A), the sample points of the H group and the C group were significantly separated in the PC1 (40.8%) and PC2 (15.8%) dimensions. Combined with the results of the permutation test (Figure 5B) (R^2^ = 0.916, Q^2^ = −0.187), the results indicate that the group differences are statistically significant and the model showed significant separation between groups. The volcano plot (Figure 5C) shows that 608 significantly downregulated metabolites and 131 significantly upregulated metabolites were identified (*p* < 0.05, |log_2_ FC| > 1). Heatmap analysis (Figure 5D) illustrated distinct metabolic profiles between the control (C) and ELE (H) groups. Hierarchical clustering of samples revealed clear segregation along the X-axis, with treatment groups forming two distinct clades, confirming that the extract reshaped the rumen metabolome. Regarding specific metabolites (Y-axis), the H group was characterized by a significant enrichment of flavonoids (e.g., Fisetin, Myricetin), phenolic acids (e.g., p-Coumaric acid, Ferulic acid), and terpenoids (e.g., Sanggenol L), which appeared as a contiguous red block indicating high relative abundance. Conversely, the C group retained higher levels of certain organic acids and amine derivatives, represented by blue hues. The color gradient (red to blue) effectively reflected the relative abundance differences between groups. The results showed that 12 metabolites were significantly enriched in the H group, including 8E-Heneicosene, SCHMEBL16385760, SCHMEBL1359410, N-acetyl zonisamide, Salicylic acid; 38 metabolites were significantly enriched in the C group, including Cadusafos, 2-Mercaptopropanoic acid/Isovalerylalanine. The top 20 bar chart of the KEGG enrichment analysis (Figure 5E) indicated that these different metabolites are significantly enriched in metabolic pathways, such as purine metabolism, β-alanine metabolism, glutathione metabolism, and antifolate resistance, cGMP-PKG signaling pathway, Olfactory transduction, PI3K-Akt signaling pathway, and MTOR signaling pathway. Finally, through the KEGG distribution map (Figure 5F), we systematically presented the distribution ratios of the differential metabolites after the intervention with Eucommia leaf extract in each KEGG pathway category. The results showed that amino acids, lipids, nucleotides, carbohydrates, signal transduction, and the endocrine system showed a downward trend. While exogenous substance degradation and terpenoid polyketide metabolism, the overall feature was that only a few pathways showed a slight upward trend.

### 3.6. Association Analysis of Significant Phenotypes, Microorganisms, and Metabolites

**Figure 6:**
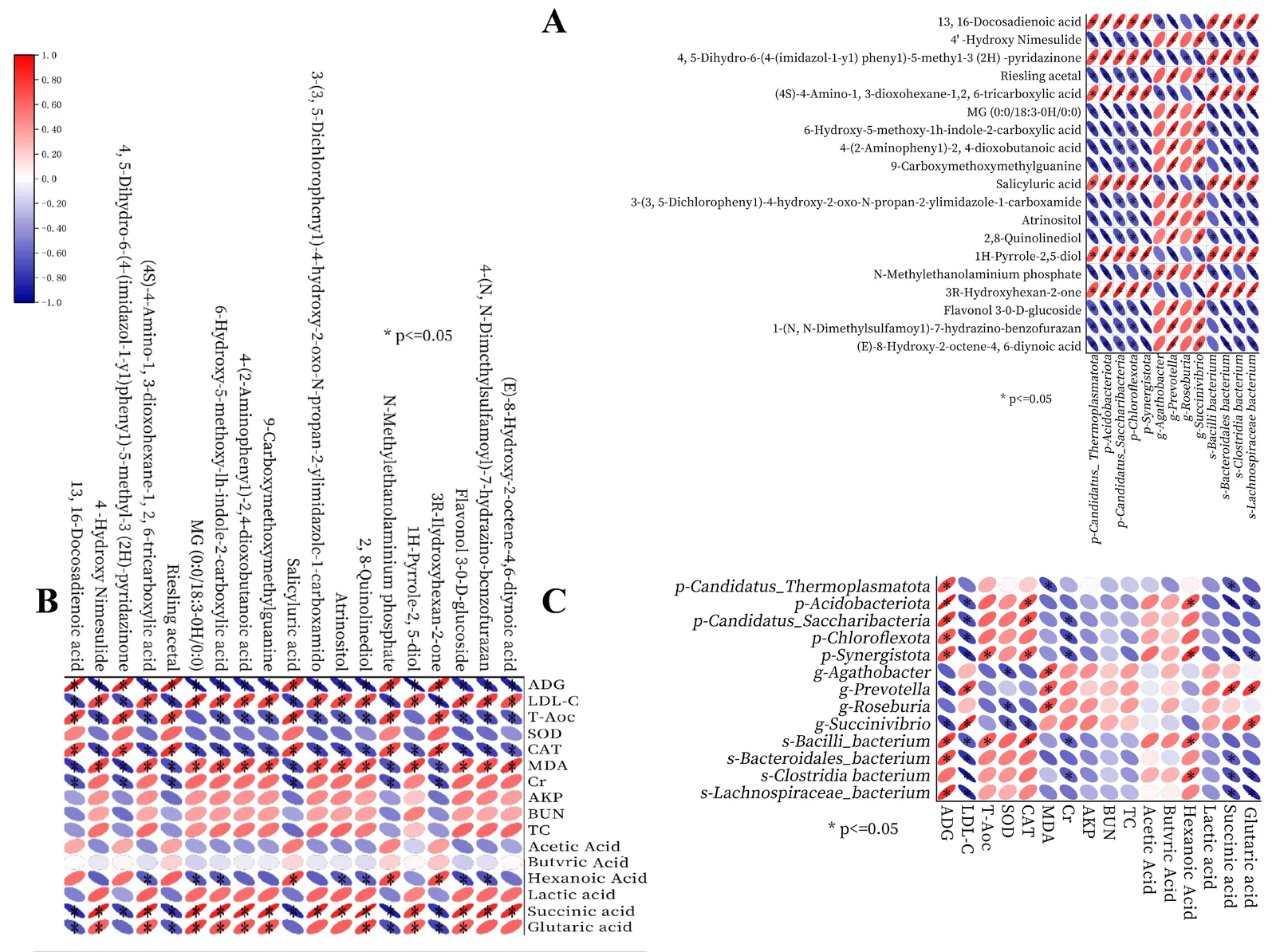
Integrated correlation heatmaps linking phenotypes, microbiota, and metabolites in yaks. (**A**) Correlation analysis between metabolites and microorganisms. (**B**) Correlation analysis between metabolites and significant phenotypes. (**C**) Correlation analysis between microorganisms and significant phenotypes. In each heatmap, red indicates a positive correlation and blue indicates a negative correlation, with color intensity proportional to the strength of the correlation; asterisks indicate statistically significant correlations.

In the correlation analysis between the metabolites and microorganisms (Figure 6A), each of the six H-enriched metabolites, namely 13,16-Docosadienoic acid, 4,5-Dihydro-6-(4-(imidazol-1-yl)phenyl)-5-methyl-3(2H)-pyridazinone, Riesling acetal, Salicyluric acid, N-Methylethanolaminium phosphate, and 3R-Hydroxyhexan-2-one, was significantly and positively correlated with every one of the nine H-enriched taxa (all 54 pairwise correlations: r = +0.64 to +0.91, all *p* ≤ 0.046) and negatively correlated with g-*Prevotella* (r = −0.78 to −0.88, all *p* ≤ 0.007) and g-*Succinivibrio* (r = −0.71 to −0.89, all *p* ≤ 0.022), whereas their correlations with g-*Agathobacter* and g-*Roseburia* were significant for only five of the 12 pairs (|r| = 0.65–0.71). Conversely, the 13 metabolites enriched in the C group were significantly and positively correlated with g-*Prevotella* (r = +0.83 to +0.91, all *p* ≤ 0.003) and g-*Succinivibrio* (r = +0.70 to +0.87, all *p* ≤ 0.025), but negatively correlated with the nine H-enriched taxa (r = −0.59 to −0.92, with 110 of the 117 pairs reaching *p* ≤ 0.05), while their correlations with g-*Agathobacter* and g-*Roseburia* were mostly non-significant (*p* ≤ 0.05 for only two of the 26 pairs). Accordingly, hierarchical clustering separated the variables into two contrasting modules, in which the H-enriched metabolites clustered with the H-enriched taxa and the C-enriched metabolites clustered with the C-enriched genera.

In the correlation analysis between the metabolites and the significantly different phenotypes (Figure 6B), the six H-enriched metabolites were positively correlated with ADG (r = +0.81 to +0.92, all *p* ≤ 0.005), T-AOC (r = +0.71 to +0.82, all *p* ≤ 0.023), and CAT activity (r = +0.69 to +0.81, all *p* ≤ 0.026), and negatively correlated with LDL-C (r = −0.71 to −0.87, all *p* ≤ 0.022), MDA (r = −0.64 to −0.83, all *p* ≤ 0.048), Cr (r = −0.66 to −0.74, all *p* ≤ 0.037), and succinic acid (r = −0.78 to −0.91, all *p* ≤ 0.008); they also showed positive correlations with hexanoic acid (r = +0.57 to +0.72, *p* ≤ 0.05 for three of the six metabolites) and negative correlations with glutaric acid (r = −0.57 to −0.73, *p* ≤ 0.05 for four of the six metabolites). In contrast, the 13 C-enriched metabolites displayed the opposite pattern, being negatively correlated with ADG (r = −0.82 to −0.94, all *p* ≤ 0.004), T-AOC (r = −0.58 to −0.70, *p* ≤ 0.05 for 11 of the 13 metabolites), CAT activity (r = −0.63 to −0.80, all *p* ≤ 0.048), and hexanoic acid (r = −0.43 to −0.73, *p* ≤ 0.05 for 9 of the 13 metabolites), and positively correlated with LDL-C (r = +0.66 to +0.82, all *p* ≤ 0.036), MDA (r = +0.64 to +0.78, all *p* ≤ 0.047), succinic acid (r = +0.76 to +0.95, all *p* ≤ 0.010), and glutaric acid (r = +0.62 to +0.75, *p* ≤ 0.05 for 8 of the 13 metabolites). No significant correlations were observed with SOD, AKP, BUN, TC, acetate, butyrate, or 2-Hydroxypropanoic acid (*p* > 0.05).

In the correlation analysis between the microorganisms and the significantly different phenotypes (Figure 6C), the H-enriched taxa were positively correlated with ADG (r = +0.64 to +0.92, *p* ≤ 0.05 for eight of the nine taxa; s-*Clostridia bacterium*, r = +0.57, *p* = 0.087) and negatively correlated with LDL-C (r = −0.65 to −0.91, *p* ≤ 0.05 for eight of the nine taxa; p-*Candidatus Thermoplasmatota*, r = −0.51, *p* = 0.126). p-*Synergistota* and s-*Bacilli bacterium* were positively correlated with T-AOC (r = +0.64 to +0.66, *p* ≤ 0.044), and p-*Acidobacteriota*, p-*Candidatus Saccharibacteria*, p-*Synergistota*, and *s-Bacilli bacterium* were positively correlated with CAT activity (r = +0.68 to +0.80, *p* ≤ 0.032). MDA was positively correlated with g-*Agathobacter*, g-*Prevotella*, *and* g-*Roseburia* (r = +0.67 to +0.72, *p* ≤ 0.033) but negatively correlated with p-*Candidatus Thermoplasmatota* (r = −0.69, *p* = 0.028). The H-enriched taxa p-*Candidatus Saccharibacteria*, p-*Chloroflexota*, p-*Synergistota*, s-*Bacilli bacterium*, and *s-Clostridia bacterium* were negatively correlated with Cr (r = −0.63 to −0.72, *p* ≤ 0.049), while p-*Acidobacteriota*, p-*Synergistota*, *s-Bacilli bacterium*, and *s-Clostridia bacterium* were positively correlated with hexanoic acid (r = +0.70 to +0.81, *p* ≤ 0.025). Succinic acid was negatively correlated with p-*Candidatus Thermoplasmatota*, p-*Acidobacteriota*, p-*Synergistota*, s-*Bacteroidales bacterium*, *s-Clostridia bacterium*, and s-*Lachnospiraceae bacterium* (r = −0.66 to −0.87, *p* ≤ 0.036) but positively correlated with g-*Prevotella* (r = +0.84, *p* = 0.002); glutaric acid showed a similar pattern, being negatively correlated with p-*Acidobacteriota*, p-*Synergistota*, *s-Clostridia bacterium*, and s-*Lachnospiraceae bacterium* (r = −0.67 to −0.86, *p* ≤ 0.035); and positively correlated with g-*Prevotella* and g-*Succinivibrio* (r = +0.71 to +0.77, *p* ≤ 0.020). In addition, g-*Prevotella* and g-*Succinivibrio* were positively correlated with LDL-C (r = +0.72 to +0.87, *p* ≤ 0.019) and negatively correlated with ADG (r = −0.74 to −0.79, *p* ≤ 0.014); g-*Agathobacter*, g-*Roseburia*, and g-*Succinivibrio* were negatively correlated with SOD (r = −0.67 to −0.68, *p* ≤ 0.034); and g-*Succinivibrio* was negatively correlated with CAT activity (r = −0.71, *p* = 0.022). No significant correlations were observed with AKP, BUN, TC, acetate, butyrate, or 2-Hydroxypropanoic acid (*p* > 0.05).

## 4. Discussion

Growth performance data demonstrated that dietary supplementation with ELE significantly elevated the ADG of yaks. These findings align with previous reports in growing pigs [30,31] and broilers [32]. In ruminants, analogous studies in finishing beef cattle [7] and sheep [10] similarly indicate that this supplementation optimizes rumen fermentation, thereby enhancing energy utilization efficiency. Furthermore, blood biochemical parameters serve as a critical proxy for evaluating the physiological status and metabolic health of livestock [26]. In monogastric animals, studies on growing pigs have confirmed that dietary supplementation with 0.50% ELE significantly enhances immune function in weaned piglets, as reported by Peng et al. [33]; this effect may be attributed to the promotion of intestinal epithelial cell repair. Additionally, flavonoid constituents within the extract exert beneficial effects on the intestinal microecology of piglets by augmenting antioxidant and anti-inflammatory activities [34]. This study demonstrated that serum T-AOC, CAT, and SOD activities were significantly elevated in the H group, whereas MDA content was markedly reduced. These findings closely align with those of Huang et al. [35] in broilers, indicating that ELE enhances systemic antioxidant enzyme activity and effectively mitigates oxidative stress. Furthermore, levels of LDL-C, TC, Cr, AKP, and BUN were significantly diminished in the H group, whereas LDL-C was significantly elevated. The reduction in TC, together with the increase in LDL-C, suggests a shift in the cholesterol distribution profile, consistent with reports by Choi et al. [36] and Yang et al. [37]. BUN and Cr serve as critical indices of protein metabolism and renal function; their decreased levels typically reflect enhanced protein utilization efficiency or attenuated catabolism, corroborating observations by Schmid et al. [38]. Modulations in AKP activity may be associated with alterations in bone metabolism or hepatic function [39,40]. Collectively, these integrated physiological improvements indicate that dietary ELE optimized protein and lipid metabolism in yaks while augmenting their capacity to withstand hypoxic oxidative stress inherent to the plateau environment, thereby providing a robust physiological foundation for the observed enhancement in growth performance.

Rumen VFAs constitute the primary energy source for ruminants, and their production profiles and molar proportions directly govern animal production performance [41,42]. Acetic acid and butyric acid serve as vital energy substrates; specifically, acetic acid acts as a precursor for lipogenesis (milk fat and body fat), whereas butyric acid is indispensable for rumen epithelial development and energy provision. In this study, the concentrations of acetic acid, butyric acid, and hexanoic acid in the rumen fluid of the H group were significantly elevated. Notably, propionic acid exhibited a very large effect size (Cohen’s d = 1.45), indicating a biologically meaningful reduction in the H group and a shift in rumen fermentation toward acetate and butyrate production. These findings align with those of previous studies [7,9,10], which reported that Eucommia leaf products improved rumen fermentation and VFA profiles in beef cattle and sheep. However, certain prior studies have yielded conclusions contrary to the present results. Notably, this study observed significantly lower concentrations of specific intermediate metabolites—namely 2-Hydroxypropanoic acid, succinic acid, and glutaric acid—in the H group. These intermediates are synthesized by specific gut microbiota via dietary fiber fermentation [43]. The ELE reshapes the rumen ecological niche via its bioactive secondary metabolites, thereby eliciting differential sensitivity responses across distinct trophic levels [44]. This process inhibits bacterial communities responsible for producing these organic acids during competition for substrate, culminating in a reduction in their biosynthesis [45]. These changes in the VFA spectra indicate that the ELE has altered the fermentation direction of rumen microorganisms, making them more inclined to produce more final products (acetic acid, butyric acid) with higher energy value, and reducing the accumulation of intermediate products that may be detrimental to the stability of the rumen environment.

Rumen microorganisms are the core driving force behind fermentation and metabolic transformation. Among them, the *Prevotella* genus is one of the most important carbohydrate-degrading bacteria in the rumen, capable of fermenting various sugars to produce succinic acid and acetic acid; the *Succinivibrio* genus is characterized mainly by the production of succinic acid [46]. This study found that *Acidobacteriota*, *p_Bacillota*, *c_Bacilli*, *Methanobrevibacter*, *Bacteroidales bacterium*, and *Lachnospiraceae bacterium* were significantly enriched in the H group. In addition, *Prevotellaceae* and *Succinivibrio* were significantly decreased. This study’s findings regarding the enrichment of the *Bacillota*/*Firmicutes* and the *Lachnospiraceae* are consistent with those reported by Sun et al. [47] in an accelerated aging mouse model. As a significant member of the *Bacillota* phylum, the *Lachnospiraceae* family is the main producer of short-chain fatty acids (especially butyric acid). The significant enrichment of *Lachnospiraceae bacterium* in this study further supports the potential mechanism by which ELE improves the host’s metabolic health by promoting the growth of butyric acid-producing bacteria [48]. Similarly, a study on broiler chickens also observed changes in the relative abundance of *Lachnospiraceae* in the cecal microbiota following ELE intervention. Moreover, it also promoted the increase in the abundance at the phylum and genus levels of Bacteroidetes [49]. In the DSS-induced colitis mouse model, the ELE reduced the abundance of the *Bacteroidaceae* family while increasing the abundance of the *Akkermansiaceae* and *Ruminococcaceae* families [50]. However, in healthy animal models, the ELE mainly demonstrated a regulatory pattern of increasing the abundance of various beneficial bacteria [51] and reducing the *Firmicutes*/*Bacteroidetes* ratio [52]. This difference mainly stems from fundamental discrepancies in the research models: in the inflammatory bowel disease model, ELE intervention aims to inhibit such bacterial communities and restore homeostasis; whereas in healthy or metabolic disorder models, the extract regulates the balance among different bacterial genera within the phylum *Bacteroidetes* through distinct pathways, rather than merely inhibiting all *Bacteroidetes* genera. It is worth noting that the significant decrease in the abundance of *Prevotella* and *Succinivibrio* in the H group suggests a shift in rumen fermentation toward a more controlled, nitrogen-efficient pattern. The decrease in *Prevotella* abundance indicates that the extract from *Eucommia ulmoides* leaves inhibits excessive protein breakdown in the rumen, thereby promoting microbial protein synthesis, reducing unnecessary NH_3_ release, and improving nitrogen utilization efficiency [53]. The decrease in *Succinivibrio* abundance in the H group indicates that the extract from *Eucommia ulmoides* leaves helps reduce postprandial fermentation spikes, thereby aiding in maintaining a more stable rumen pH and redirecting carbon flux toward a more balanced VFA profile [54]. Furthermore, this study observed the enrichment of the methanogen *Methanobrevibacter* in the H group. The changes in its abundance are associated with host digestive efficiency and energy acquisition [55]. The enrichment might be due to the fact that the extract from *Eucommia ulmoides* interfered with the fermentation pattern and redox state of the intestinal tract. Similarly, in sheep, it was also found that the ELE altered the rumen microbial composition and increased the relative abundance of cellulose-decomposing bacteria [10], which appears contrary to conclusions regarding the performance improvement of weaned piglets by ELE [33]. It is worth noting that in the H group, the abundance of *Bacillus* species was significantly increased. This is because the extract from *Eucommia ulmoides* leaves contains specific polysaccharide components [56]. Studies have shown that members of the phylum *Acidobacteriota* possess abundant carbohydrate-active enzymes, enabling efficient degradation of complex plant-derived polysaccharides [57]. This shift in the microbial community aligns with previous findings that herbal polysaccharides can reshape the gut ecosystem toward a glycolytic fermentation profile [58]. This indicates that under specific pathological conditions, ELE primarily exerts its effects by modulating bacterial communities associated with dysbiosis. We conclude that the extract reduces nutrient competitive pressure in the rumen by inhibiting dominant bacteria (e.g., *Prevotella*), thereby affording ecological niches for low-abundance taxa possessing specific metabolic functions. This may represent the microbial basis for the enhanced fiber digestibility observed with ELE.

Alterations in microbial community structure are invariably accompanied by shifts in functional potential [59]. In this study, the H group showed enrichment of pathways related to genetic regulation, metabolism, and cellular signaling, consistent with the documented capacity of *ELE* to protect against chemical-induced oxidative stress and DNA damage [60]. Recent investigations further demonstrate that its bioactive ingredients exhibit potent antioxidant properties [61]. These findings closely align with prior reports indicating that ELE reduces 8-OHdG production and mitigates DNA strand breaks [62]. Meanwhile, the significant enrichment of the “transcriptional machinery” and “translation machinery” pathways indicates that the regulatory effect of ELE on fundamental cellular processes extends beyond the DNA level to encompass multiple signal transduction cascades and the modulation of transcription factor expression [63]. The putative underlying mechanism involves the scavenging of free radicals and activation of the Nrf2/ARE signaling axis, thereby permitting cellular resource reallocation toward canonical gene expression and protein synthesis programs [63,64]. Conversely, investigations employing metabolic dysfunction models (e.g., hyperlipidemia) more frequently document the capacity of ELE to modulate lipid metabolism [65,66] and amino acid catabolism [67]. This indicates that ELE can induce the microbial community to enter a state of active proliferation and stress adaptation. This disparity likely reflects the high model-dependency of ELE in modulating microbial community function: under basal physiological conditions, it primarily influences nutrient metabolism; whereas under specific interventions (e.g., this experiment), it may predispose the community to activate genetic maintenance mechanisms to cope with environmental perturbations.

This study demonstrated that although the H group exhibited enrichment of genes such as rpoB2 and rpoB, the overall antimicrobial resistance profile shifted from “high-risk tetracycline target modification” toward mechanisms including “multidrug efflux.” Analogous remodeling of multidrug resistance genes was previously reported for allicin intervention [68]. Notably, virulence factors were broadly diminished in the H group, while core glycolytic enzymes (GT4, CE1) remained stable. Consistent with findings [51], ELE optimizes intestinal microbiota structure and enhances host resilience. The underlying mechanism of this beneficial modulatory effect involves suppressing the proliferation of pathogenic bacteria and their virulence expression. Furthermore, reports confirm that ELE reshapes rumen and gut microecology, mitigates disease risk, and improves metabolic efficiency [69]. This implies that the extract may alter the host-associated reservoir of antimicrobial resistance genes via mechanisms akin to those described, thereby reducing the potential for horizontal transfer of resistance determinants [70].

This study, via non-targeted metabolomics profiling, revealed that dietary ELE induced extensive metabolic reprogramming within the yak rumen, with 608 metabolites exhibiting significant downregulation. This finding directly indicates that the bioactive plant secondary metabolites (e.g., polyphenols and flavonoids) inherent to the extract exert a profound modulatory impact on the rumen microecosystem [44]. Unlike conventional nutrients that broadly fuel anabolism, polyphenols exert their biochemical effects primarily through protein binding and differential antimicrobial pressure, selectively suppressing specific microbial guilds and enzymatic activities without indiscriminately stimulating biosynthesis [71]. Consequently, the majority of the downregulated metabolite pool clustered within amino-acid-derived and peptidolytic-associated nodes, reflecting the established capacity of polyphenols to mitigate proteolytic deamination and ammonia release via protein–polyphenol complexation and the inhibition of peptidolytic enzymes [45]. Notably, a generalized downregulation was observed across core metabolic pathways, including amino acid, lipid, and nucleotide metabolism, as well as the PI3K-Akt/mTOR signaling cascade, primarily reflecting the inhibitory impact of ELE on targeted rumen microbial consortia [44]. This phenomenon aligns with prior reports demonstrating that ELE mitigates metabolic stress and enhances barrier function [56]. Concurring evidence indicates that plant extracts modulate nitrogen metabolism via the gut microbiota [58], a finding consistent with the observed downregulation of purine and β-alanine metabolism in this study [71]. Previous studies confirm that plant polyphenols selectively inhibit specific rumen Gram-positive bacteria, thereby redirecting microbial metabolic flux and reducing the turnover of amino acids and nucleotides [44]. The suppression of lipid metabolism may reflect interference by ELE with microbial fatty acid hydrogenation pathways [44]. Furthermore, given the established antioxidant and anti-inflammatory properties of ELE, the downregulation of the PI3K-Akt/mTOR axis likely signifies attenuated local inflammatory tone within the rumen [72]. In the harsh, hypoxic environment of the Qinghai–Xizang Plateau, rumen tissues of yaks are persistently challenged by oxidative stress [73]. Inhibiting this pathway constitutes a pivotal mechanism whereby ELE confers cytoprotection and preserves the structural integrity of the rumen epithelial barrier [50]. In summary, ELE transforms the rumen from a “high metabolic burden, high signal activation” stress state to a “high metabolic efficiency, homeostatic maintenance” healthy state [56,69].

Integrating the correlation analyses, this study demonstrates that alterations in the rumen microbiota and metabolome are intimately linked to improvements in host phenotypic parameters, revealing an extensive network of potential interactions. Microbial taxa enriched in the ELE group exhibited positive correlations with ADG and systemic antioxidant indices. Concurrently, metabolites positively associated with growth and health trajectories were predominant in the treatment group and correlated with specific beneficial microbial consortia. This indicates that the extract does not merely alter isolated indicators; rather, it systematically reshapes the intricate network wherein the structural configuration and metabolic output of the rumen microbiome govern host physiological and biochemical parameters, ultimately enhancing production performance. Within this framework, shifts in microbial community architecture serve as the impetus for subsequent functional and metabolic adaptations, while the optimization of the rumen fermentation profile (VFA profile) and blood biochemical parameters constitute the pivotal intermediaries linking microbial modulation to improved host growth performance.

Several limitations of this study should be acknowledged. Dry matter intake (DMI) was not measured during the experimental period; since ADG can also be influenced by DMI, the observed improvement in growth performance cannot be attributed solely to ELE supplementation. In addition, the determination of live weight in ruminants is influenced by factors such as gastrointestinal fill and the timing of weighing relative to feeding, and invasive sampling (blood and rumen fluid) was restricted to a randomly selected sub-sample of animals (*n* = 5). Future studies incorporating DMI recording and larger sample sizes are warranted to corroborate these findings.

## 5. Conclusions

In summary, dietary supplementation with ELE significantly increased ADG, T-AOC, CAT, SOD, and the rumen concentrations of acetic, butyric, and hexanoic acids. Conversely, it significantly decreased serum MDA, Cr, AKP, BUN, and TC, and significantly increased LDL-C, as well as rumen lactic, succinic, and glutaric acids. This study further revealed that the H group was significantly enriched in *Acidobacteriota*, *Bacillota*, *Bacilli*, *Methanobrevibacter*, *Bacteroidales bacterium*, and *Lachnospiraceae bacterium*, whereas the abundances of *Prevotella*, *Succinivibrio*, and the functional profiles of VFDB and CAZy were markedly reduced. Additionally, the number of downregulated metabolites substantially exceeded that of upregulated metabolites. Collectively, these findings provide a theoretical basis and practical reference for the application of ELE as a potential feed additive in yak production systems. However, given the limitations of this study (small sample size, single dosage level, and lack of individual DMI measurement), further research with larger cohorts and multiple supplementation levels is warranted before broad practical application can be recommended.

## Figures and Tables

**Table 1:** Ingredient composition and nutrient levels (dry matter basis).

Ingredients, %	Percentage	Nutrient Levels	Content
Corn (8.0% CP)	58.75	Crude protein, %	19.47
Corn bran (10.0% CP)	6.00	Rumen bypass protein, %	14.80
Molasses (5.5% CP) ^1^	3.00	Rumen degradable protein, %	16.61
Cottonseed meal (46.0% CP)	12.00	Soluble protein, %	19.07
Soybean meal (44.0% CP)	12.90	Metabolizable energy, MJ/kg	12.66
Dried vinasse (28.0% CP)	2.00	Net energy, MJ/kg	8.16
Fine ground limestone	1.50	Maintenance net energy, MJ/kg	8.16
Calcium hydrogen phosphate	0.80	Net energy gain, MJ/kg	5.83
Feed grade sodium chloride	1.20	Acid detergent fiber, %	9.55
Sodium bicarbonate	0.60	Neutral detergent fiber, %	18.90
Feed grade magnesium oxide	0.20	Effective neutral detergent fiber, %	5.74
1% Feed premix ^2^	1.00	Non-fiber carbohydrate, %	52.85
Ruminant Flavor 161K ^3^	0.02	Silage acid, %	0.23
Acetohydroxamic acid	0.02	Soluble sugar, %	6.72
Deodorant	0.01	Starch, %	40.86
Total	100.00	Soluble fiber, %	5.03
		Total crude fat, %	2.72
		Long chain fatty acid, %	2.65
		Crude ash, %	9.29
		Ca, %	0.95
		P, %	0.65
		Mg, %	0.36
		K, %	0.96
		S, %	0.24
		Na, %	0.84
		Cl, %	0.94
		Lignin, %	1.43

Note: ^1^ The crude protein content of molasses was referenced as 5.5% CP on a dry matter basis per Feedipedia [15], and all protein-related nutrient values in Table 1 have been recalculated accordingly. ^2^ The constituents of each kilogram of feed premix are as follows: Vitamin A 3500 IU, Vitamin D 1000 IU, Vitamin E 40 IU, Mn 40 mg, Fe 50 mg, Cu 10 mg, Zn 40 mg, Se 0.3 mg. ^3^ Ruminant Flavor 161K was a feed attractant added to improve palatability. Dried vinasse was a byproduct of yeast fermentation, primarily consisting of metabolites from *Saccharomyces cerevisiae*.

**Table 2:** Effect of ELE on the growth performance of yaks (*n* = 5).

Items	Groups		*p*-Value
	C Group	H Group	
Initial body weight (IBW), kg	153.48 ± 7.48	146.72 ± 1.87	0.406
30d body weight (30d BW), kg	155.01 ± 5.24	154.42 ± 1.67	0.919
Final body weight (FBW), kg	167.09 ± 3.15	169.01 ± 3.38	0.689
Average daily gain (ADG), kg/d	0.23 ± 0.01	0.33 ± 0.01	<0.001

Note: Group comparisons were performed using independent samples *t*-tests. Values are mean ± SEM. Homogeneity of variances was confirmed by Levene’s test for all parameters (*p* > 0.05).

## Data Availability

Sequencing datasets are available in the NCBI database under accession number PRJNA1462487.

## Supplementary Materials

The following supporting information can be downloaded at: https://www.mdpi.com/article/10.3390/ani16182965/s1, Table S1: Sequencing quality statistics of rumen metagenomic samples from yaks in the control group (C) and the Eucommia leaf extract-supplemented group (H); Table S2: Assembly statistics of contigs obtained from rumen metagenomic sequencing of each yak in the C and H groups; Table S3. Prediction statistics of open reading frames (ORFs) identified in the rumen metagenomes of each yak in the C and H groups.

## Abbreviations

The following abbreviations are used in this manuscript:

ADG	Average Daily Gain
AST	Aspartate Aminotransferase
BUN	Blood Urea Nitrogen
CARD	Comprehensive Antibiotic Resistance Database
CAT	Catalase
CAZY	Carbohydrate-Active enZYmes Database
Cr	Creatinine
ELE	*Eucommia ulmoides* leaf extract
GSH-Px	Glutathione peroxidase
HDL-C	High-Density Lipoprotein Cholesterol
KEGG	Kyoto Encyclopedia of Genes and Genomes
LDL-C	Low-Density Lipoprotein Cholesterol
MDA	Malondialdehyde
SOD	Superoxide dismutase
T-AOC	Total Antioxidant Capacity
TC	Total Cholesterol
VFDB	Virulence Factor Database
