# Supplementary material for: Effects of Eucommia ulmoides Leaf Extract on Growth Performance, Serum Biochemistry, Rumen Microbiota, and Metabolic Profiles in Yaks
Source: Animals (Basel). 2026 Sep 20;16(18):2965. doi: 10.3390/ani16182965 (PMC13603303; doi:10.3390/ani16182965)
Supplement: Supplementary file 1 [file animals-16-02965-s001.zip › animals-4435674-supplementary.pdf]

Table S1. Sequencing quality statistics of rumen metagenomic samples from yaks in the control group (C) and the Eucommia leaf extract-supplemented group (H)

Sample	Raw Reads	Clean Reads	Clean Reads (%)	GC (%)	Q20(%)	Q30(%)
C1	78648952	78620522	99.96%	45.54%	98.94%	96.61%
C2	81663040	81637794	99.97%	45.85%	98.95%	96.52%
C3	76690978	76657368	99.96%	44.66%	98.91%	96.50%
C4	79726594	79695636	99.96%	45.50%	98.98%	96.63%
C5	82099150	82066848	99.96%	45.58%	98.91%	96.49%
H1	80721570	80688250	99.96%	43.06%	98.94%	96.56%
H2	76109412	76074054	99.95%	46.25%	98.51%	95.09%
H3	78254090	78223228	99.96%	43.37%	98.97%	96.67%
H4	78936580	78858440	99.90%	46.51%	98.98%	96.74%
H5	77953530	77915114	99.95%	44.25%	98.94%	96.62%

[1] Q20 (%): The percentage of bases with a base recognition accuracy of more than 99%;

[2] Q30 (%): The percentage of bases with a base recognition accuracy of more than 99.9%.

Table S2. Assembly statistics of contigs obtained from rumen metagenomic sequencing of each yak in the C and H groups

Sample	Total Size(bp)	Contigs Num	Mean Len	Max Len	Min Len	GC (%)	N50	N90
C1	398513567	316471	1259.242	436947	500	45.70%	1399	581
C2	412642755	262837	1569.957	555083	500	45.30%	2246	619
C3	418000251	291087	1435.998	616990	500	44.68%	1851	600
C4	479951069	353876	1356.269	476154	500	45.51%	1614	593
C5	489879157	361862	1353.773	632723	500	45.60%	1612	593
H1	659633630	478222	1379.346	386482	500	42.29%	1690	596
H2	553335650	401284	1378.913	402016	500	45.45%	1684	607
H3	549695249	392040	1402.141	433281	500	41.89%	1716	596
H4	506145525	397619	1272.941	354292	500	47.88%	1448	579
H5	638119630	538679	1184.601	400589	500	44.37%	1283	574

[1] N50: The number of contigs longer than N50 accounts for 50% of the total;

[2] N90: The number of contigs longer than N90 accounts for 90% of the total.

Table S3. Prediction statistics of open reading frames (ORFs) identified in the rumen metagenomes of each yak in the C and H groups

Sample	Total Size	ORF Num	Mean Len	Max Len	Min Len	GC (%)
C1	348969528	502914	693.895	27876	201	46.71%
C2	364668594	472189	772.2937	55170	201	46.30%
C3	370954605	495828	748.1518	21180	201	45.56%
C4	423967452	581265	729.3875	25989	201	46.49%
C5	432926253	594356	728.3955	27876	201	46.58%
H1	582303633	791986	735.2449	25467	201	43.16%
H2	488601588	688541	709.6187	22977	201	46.43%
H3	483462051	667888	723.867	31389	201	42.79%
H4	452926641	620872	729.5008	30996	201	48.80%
H5	563270433	823371	684.1028	31389	201	45.32%
